# Nontuberculous Mycobacterial Parotid Abscess: Clinical Evaluation and Management of a Rare Case in an Adult Female With Sjögren’s Syndrome

**DOI:** 10.7759/cureus.100752

**Published:** 2026-01-04

**Authors:** Adeeba F Ghias, Michael Black, Jae Lim

**Affiliations:** 1 Otolaryngology - Head and Neck Surgery, Tripler Army Medical Center, Honolulu, USA; 2 Pathology, Kaiser Permanente Moanalua Medical Center, Honolulu, USA; 3 Otolaryngology - Head and Neck Surgery, Hawaii Permanente Medical Group, Kaiser Permanente Moanalua Medical Center, Honolulu, USA; 4 Otolaryngology - Head and Neck Surgery, John A. Burns School of Medicine, University of Hawaii, Honolulu, USA

**Keywords:** adult atypical mycobacterium, immunocompetent, mycobacteria, parotid abscess, parotitis

## Abstract

This report presents a case of a young female with Sjögren’s disease who developed a non-tuberculous mycobacterial (NTM) abscess in the right parotid gland. Following an unsuccessful four-month course of antibiotic therapy, she underwent a superficial parotidectomy. This was an unusual case in this demographic, considering that most cervical NTM infections are diagnosed in pediatric patients who have a characteristic violaceous neck mass. Her case was reviewed using the medical record, and photo-documentation was conducted throughout follow-up visits in the outpatient clinic. Infectious disease specialists were heavily involved in determining her antibiotic treatment plan, which spanned over four months. The initial therapy was complicated by multiple antibiotic changes and pauses in treatment due to adverse effects, including chest tightness and elevation of liver function tests (LFTs). After completion of antibiotic therapy, the patient continued to experience episodes of parotitis without abscess, and she ultimately underwent a right superficial parotidectomy. No evidence of recurrence was observed during postoperative follow-up. This case report illustrates a rare cause of NTM infection in an immunocompetent adult patient and provides important management guidance for providers who may encounter similar patients.

## Introduction

Parotitis, as a broad term, encompasses multiple possible etiologies of parotid disease, whether infectious or non-infectious. The mumps virus, Staphylococcal species, *Streptococcus viridans*, and other anaerobic oral flora are commonly implicated [1]. However, few existing reports have mentioned the incidence of infection with non-tuberculous mycobacterial (NTM) species in an immunocompetent host. The most common presentation of NTM in the head and neck region is a violaceous neck mass in the pediatric population, caused by the Mycobacterium avium-intracellulare complex (MAC) [2-4]. The transmission pattern is thought to be through soil exposure [5]. Multi-drug therapy is often required and is described in the pediatric infectious disease literature, involving combinations of azithromycin or clarithromycin, clofazimine, ethambutol, isoniazid, rifampin, and/or amikacin [2,3]. The treatment course duration is highly dependent on the response to initial antimicrobial therapy [2].

## Case presentation

**Figure 1:**
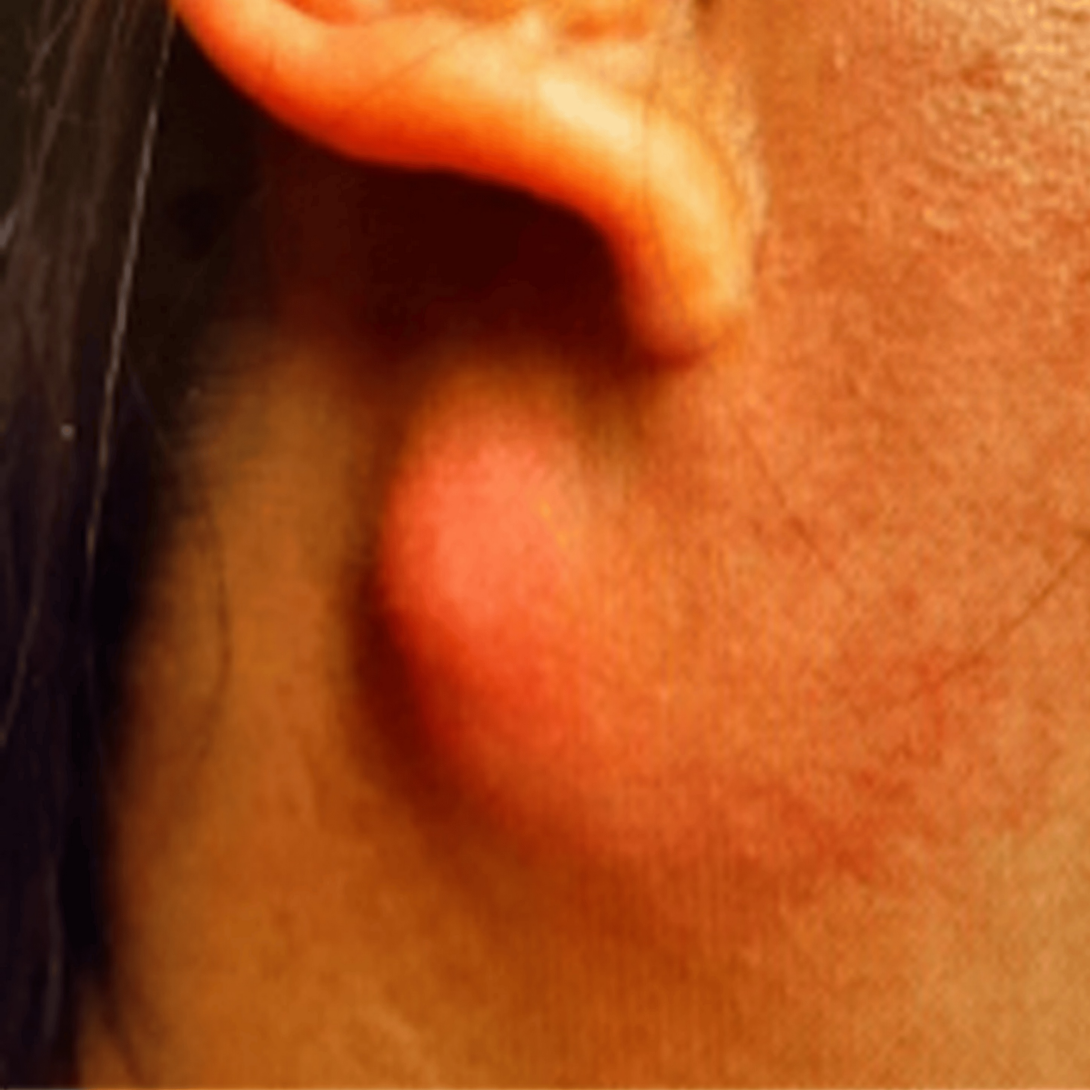
Physical examination during active infection.

We present a case of a 38-year-old Asian female with a history of Sjögren’s disease, asthma, alpha-thalassemia trait, and non-alcoholic steatohepatitis who had previous intermittent episodes of right parotid swelling and then presented to the emergency room with a fluctuant, multiloculated right parotid abscess extending nearly through the epidermis (Figure 1). On history, she had never been on immunosuppressive medications, nor was she ever in an immunocompromised state. She reported no sick contacts and no recent travel. Her facial nerve function remained intact despite the presenting complaint. The abscess was drained, cultured, and the resultant wound packed with iodoform; the patient was discharged on clindamycin and a steroid taper until her short-interval outpatient follow-up for removal and replacement of packing. Despite a good interval result at a follow-up visit, she started to develop increasing pain on the last day of her week-long antibiotic course. Sulfamethoxazole-trimethoprim was then added. Wound cultures that were initially negative then grew 2+ highly resistant strains of Mycobacterium abscessus. Appropriate infectious disease follow-up was arranged. She continued to have drainage from the site despite additional antibiotic treatment, and there was concern for recollection of abscess as well as fistulization. She underwent surgical incision and drainage with drain placement in the operating room 20 days after initial presentation.

**Figure 2:**
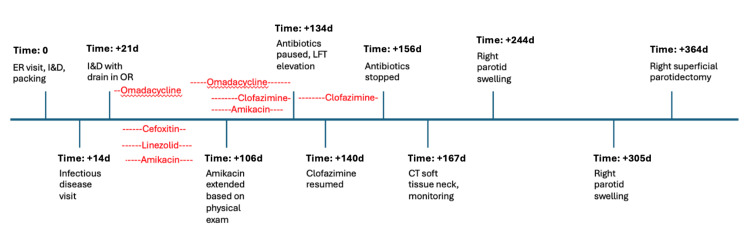
Chronological timeline of key events during treatment, with antibiotics superimposed to clarify the timing of administration. Please note that the initial “Time = 0” point is followed by progressively increasing time (in days) from the ER visit. LFT: Liver function test; I&D: Incision and drainage.

**Figure 3:**
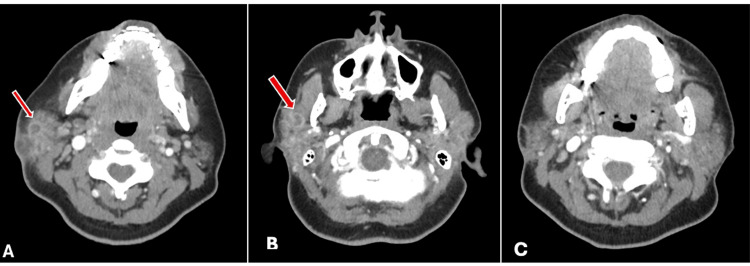
CT soft tissue neck scan showing (A) the initial rim-enhancing infection pre-treatment, (B) recurrence of symptoms and radiologic findings at five months, and (C) post-treatment following antibiotic therapy and superficial parotidectomy.

Per our infectious disease colleagues, this was a highly resistant bacterium requiring likely four to six weeks of combination IV and PO multiagent antibiotic therapy. Bacterial growth occurred from culture at eight weeks, in a delayed fashion, as is typical of NTM infections. Initial data indicated susceptibility to amikacin and intermediate susceptibility to cefoxitin, imipenem, and linezolid. Resistance was noted to ciprofloxacin, clarithromycin, doxycycline, moxifloxacin, and sulfamethoxazole-trimethoprim. These data were used to guide the treatment plan. Her complicated antibiotic course and medical timeline are detailed in Table 1 and Figure 2. Clofazimine was initiated as a study drug, so there was a delay in obtaining it at our institution. Audiology performed appropriate ototoxic monitoring while on an aminoglycoside (amikacin), with high-frequency audiologic testing and otoacoustic emissions testing (OAEs). A follow-up CT soft tissue scan of the neck was conducted five months after her initial presentation to help guide antibiotic duration. This scan demonstrated near-resolution of her abscess, and therefore, all antibiotics were discontinued after the patient completed eighteen weeks of treatment. Note that her treatment course was not truly continuous due to the adverse effects as listed in Table 1. Figure 3 demonstrates her juxtaposed initial presentation and post-treatment resolution. She had recurrence of parotid swelling without abscess three months and then five months later. These presentations were treated with a steroid taper, once with amoxicillin-clavulanate and once with linezolid, respectively. The swelling resolved, although a five-month post-treatment scan demonstrated recurrence of two intraparenchymal fluid collections measuring one centimeter and half a centimeter.

**Table 1:** Summary of antibiotics received by the patient after initial one-week therapy with clindamycin, followed by nearly one week each of vancomycin, ampicillin-sulbactam, and sulfamethoxazole-trimethoprim. Note: These medications were administered prior to availability of susceptibility results. Antibiotics were changed throughout her treatment course due to adverse effects. BID: Twice daily; IV: Intravenous; PICC: Peripherally inserted central catheter; LFT(s): Liver function test(s).

Antibiotic	Dose (mg)	Duration	Adverse effects
Omadacycline	450 BID → 300 daily	Cycle 1: 23 days; Cycle 2: 85 days	-
Clofazimine (study drug)	100 daily	Cycle 1: 70 days; Cycle 2: 17 days	LFT elevation to 3× upper limit of normal
Amikacin	700 mg IV daily via PICC	Cycle 1: 25 days; Cycle 2: 62 days	-
Linezolid	600 daily	25 days	-
Cefoxitin	3000 BID via PICC	14 days	Chest tightness
Imipenem	1000 BID via PICC	7 days	-
Vancomycin	N/A (given perioperatively / as a bridge to above)		
Ampicillin-sulbactam			
Sulfamethoxazole-trimethoprim			

**Figure 4:**
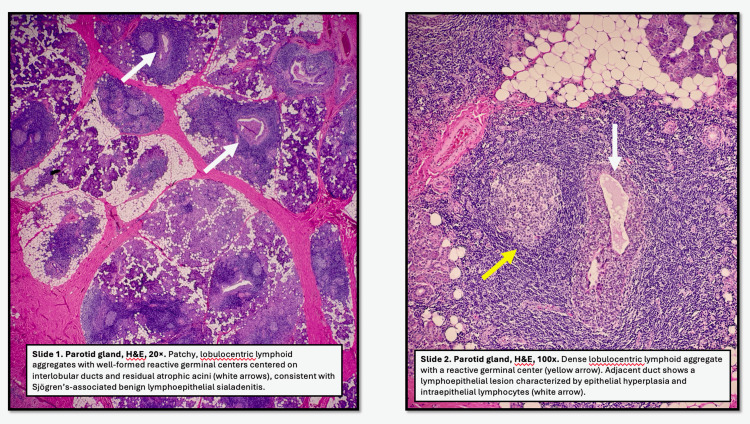
Histopathological examination from the patient’s superficial parotidectomy, provided by the pathologist, Dr. Michael Black. (A) Parotid gland, H&E, 20×. Patchy, lobulocentric lymphoid aggregates with well-formed reactive germinal centers centered on interlobular ducts and residual atrophic acini, consistent with Sjögren’s-associated benign lymphoepithelial sialadenitis. (B) Parotid gland, H&E, 100×. Dense lobulocentric lymphoid aggregate with a reactive germinal center. The adjacent duct shows a lymphoepithelial lesion characterized by epithelial hyperplasia and intraepithelial lymphocytes.

Given the recurrent symptoms, a superficial parotidectomy was recommended. The patient consented to the procedure and, for aesthetic contouring, concurrently underwent a sternocleidomastoid muscle rotation flap and abdominal fat grafting to the site. Final histopathologic analysis (Figure 4) and culture did not reveal any atypical mycobacterial growth. Final pathology reported “abscess, features consistent with Sjögren syndrome, and two benign intraparotid lymph nodes.” Her operative site has healed well, and there has been no further recurrence of symptoms. 

## Discussion

NTM cervical lymphadenitis is classically described in the pediatric population, with a slight female-to-male predominance [3]. Of the NTM infections, Mycobacterium abscessus is characterized by its slow growth in culture media [2]. Therefore, the initial diagnosis of atypical mycobacterial infections can be difficult and is subsequently complicated by multidrug resistance [6]. Although in this case the cause of the patient’s parotid swelling was deemed infectious, a neoplasm must be ruled out in the evaluation process [6]. Imaging findings are non-specific, with these abscesses appearing as cystic nodal masses that do not cause mass effect or significant fat stranding in the surrounding tissue [7]. Other important differential diagnoses to consider are toxoplasmosis, cytomegalovirus (CMV) or Epstein-Barr virus (EBV) infection, sarcoidosis, and Bartonella [3,8].

There are two reports of parotid abscesses in immunocompetent adult patients, one of a 48-year-old female [9] and the other in a 79-year-old male [5]. The female patient had a multi-year history of suppurative bacterial sialadenitis prior to diagnosis of Sjögren’s disease, later necessitating parotidectomy [9]. Underlying autoimmune parotid disease, like Sjögren’s, although known to cause recurrent sialadenitis and intermittent recurrent swelling of the major salivary glands, does not have a strong association with an increased risk of abscess formation or atypical infection [9]. The male patient required eight months total of various antibiotics for cure of his NTM parotid abscess without the need for parotidectomy, and he had no evidence of recurrence through four years of surveillance [5].

The loose treatment algorithm accepted in the pediatric literature recommends initial antimicrobial trials to avoid unnecessary and disfiguring surgical intervention. A combination of national and international studies report a variable response rate between patients in the United States (50%), the Netherlands (66%), and France (90%) after studying multiple case series at their respective pediatric hospitals [2,3,10]. As interpreted from the available literature, antibiotic therapy alone has demonstrated a lower rate of cure than antibiotic therapy in combination with surgery and should be reserved for cases where medical comorbidities prevent surgical intervention or where the patient adamantly refuses surgery [2-4]. Due to the adverse effect profiles and poor compliance with the long duration of the antibiotics utilized in NTM treatment, the literature has historically favored surgical excision [2,6]. Commonly cited drug effects are weight loss, gastrointestinal upset, nausea, and allergic reactions [2]. In fact, Lindeboom et al [10] published a 78% adverse effect rate in their study compared to a 28% surgical complication rate. There is consensus within the literature that if operative intervention is required, excision is preferred over incision and drainage to reduce the risk of relapse and/or fistulization to the skin [2,3,8]. Preoperative medical therapy, by decreasing edema within the salivary gland, decreases the risk of subsequent surgical morbidity, particularly facial nerve injury [11].

Involvement of infectious disease specialists is critical in drug administration, titration, and monitoring treatment response [2]. Clarithromycin is most frequently cited in the literature, combined synergistically with ethambutol or rifabutin [3,11]. However, there is no standardization of antibiotic duration or timing of surgery in cases of NTM salivary gland abscesses, with the literature reporting multiple different combinations of antibiotics as well as a range of therapy from a minimum of two weeks to up to eight months [3-5,11].

## Conclusions

Although the existing literature provides limited evidence and lacks a well-established consensus on the occurrence of NTM parotid abscess in patients with Sjögren’s syndrome, this patient achieved complete resolution of her atypical mycobacterial parotid abscess after approximately one year of combined medical and surgical management. A multimodal therapeutic approach is supported by multiple clinical reports, despite significant variation in the duration of antimicrobial therapy and the choice of drug regimens. Individualized treatment planning is critical and should be guided by a thorough evaluation of the risks and benefits associated with long-term antimicrobial therapy compared to surgical intervention for each individual case. Additional case reports and systematic studies of this rare condition are necessary to establish robust, evidence-based treatment algorithms and improve long-term clinical outcomes.

## References

[1] Thiede O, Stoll W, Schmäl F (2002). [Clinical aspects of abscess development in parotitis]. HNO.

[2] Berkovic J, Vanchiere JA, Gungor A (2016). Non tuberculous mycobacterial lesion of the parotid gland and facial skin in a 4year old girl: a proposed treatment strategy. Am J Otolaryngol.

[3] Rives P, Joubert M, Launay E, Guillouzouic A, Espitalier F, Malard O (2016). Cervicofacial non-tuberculous mycobacteria: a report of 30 cases. Eur Ann Otorhinolaryngol Head Neck Dis.

[4] Tunkel DE (1995). Atypical mycobacterial adenitis presenting as a parotid abscess. Am J Otolaryngol.

[5] Yamanaka T, Okamoto H, Hosoi H (2013). Non-tuberculous mycobacterial infection of the parotid gland in an immunocompetent elderly patient. BMJ Case Rep.

[6] Holmes S, Gleeson MJ, Cawson RA (2000). Mycobacterial disease of the parotid gland. Oral Surg Oral Med Oral Pathol Oral Radiol Endod.

[7] Bagla S, Tunkel D, Kraut MA (2003). Nontuberculous mycobacterial lymphadenitis of the head and neck: radiologic observations and clinical context. Pediatr Radiol.

[8] Stanley RB, Fernandez JA, Peppard SB (1983). Cervicofacial mycobacterial infections presenting as major salivary gland disease. Laryngoscope.

[9] Knopf A, Pickhard A, Stark T, Schulz S, Scherer EQ (2009). [Recurrent abcesses of the parotid gland in Sjögren's syndrome]. HNO.

[10] Lindeboom JA, Kuijper EJ, Bruijnesteijn van Coppenraet ES, Lindeboom R, Prins JM (2007). Surgical excision versus antibiotic treatment for nontuberculous mycobacterial cervicofacial lymphadenitis in children: a multicenter, randomized, controlled trial. Clin Infect Dis.

[11] Shah MB, Haddad J Jr (2004). Nontuberculous mycobacteria-induced parotid lymphadenitis successfully limited with clarithromycin and rifabutin. Laryngoscope.

